# Supplementary material for: Splice-Junction-Based Mapping of Alternative Isoforms in the Human Proteome
Source: Cell Rep. Author manuscript; Available in PMC 2020 Jan 15. (PMC6961840; doi:10.1016/j.celrep.2019.11.026)
Supplement: 2 [file NIHMS1546469-supplement-2.zip › DF1/PXD006675/RightVentricle/RightVentricle_40_OTUD5_GALVPPPDADPANEPPPPGPMPPAPR.pdf]

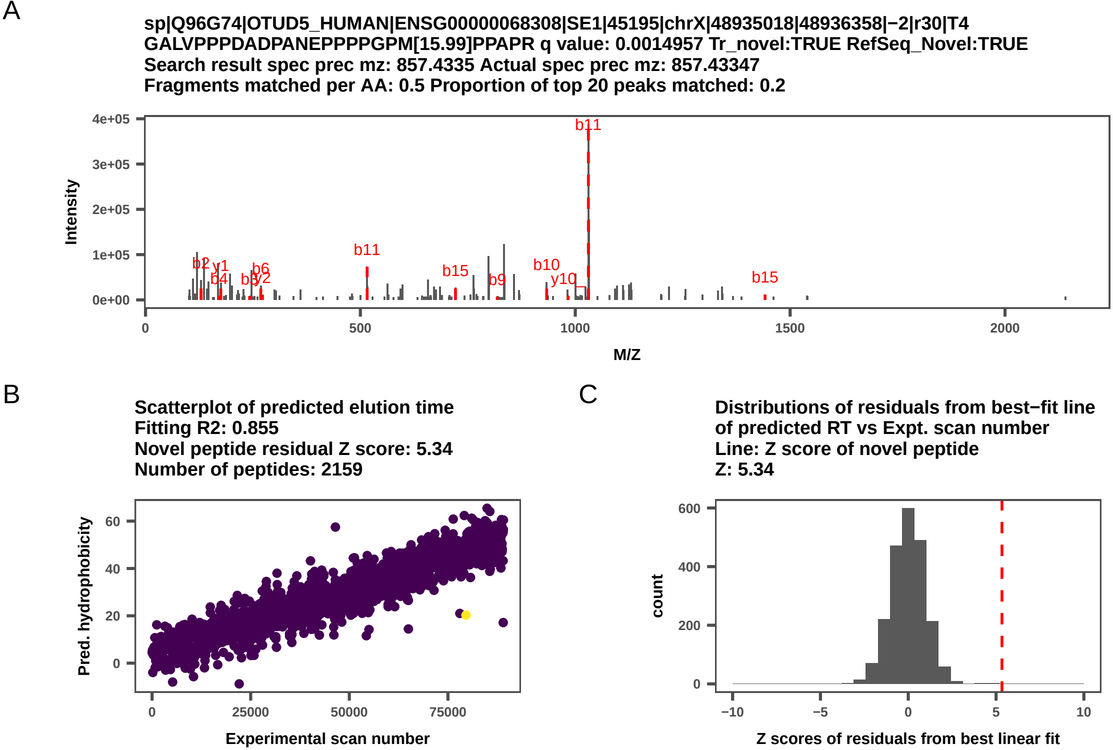
A
sp|Q96G74|OTUD5_HUMAN|ENSG00000068308|SE1|45195|chrX|48935018|48936358|−2|r30|T4
GALVPPPDADPANEPPPPGPM[15.99]PPAPR q value: 0.0014957 Tr_novel:TRUE RefSeq_Novel:TRUE
Search result spec prec mz: 857.4335 Actual spec prec mz: 857.43347
Fragments matched per AA: 0.5 Proportion of top 20 peaks matched: 0.2
Intensity
4e+05
3e+05
2e+05
1e+05
0e+00
b11
b2
y1
b4
b6
b3
y2
b11
b15
b9
b10
y10
b15
0
500
1000
1500
2000
M/Z
B
Scatterplot of predicted elution time
Fitting R2: 0.855
Novel peptide residual Z score: 5.34
Number of peptides: 2159
Pred. hydrophobicity
60
40
20
0
0
25000
50000
75000
Experimental scan number
C
Distributions of residuals from best−fit line
of predicted RT vs Expt. scan number
Line: Z score of novel peptide
Z: 5.34
count
600
400
200
0
−10
−5
0
5
10
Z scores of residuals from best linear fit
